# Autotransplantation of Two Immature Third Molars with the Use of L-PRF

**DOI:** 10.1155/2021/6672711

**Published:** 2021-01-02

**Authors:** Manon Rey Lescure, Nicola Alberto Valente, Sibylle Chatelain, Chiara Cinquini, Antonio Barone

**Affiliations:** ^1^Unit of Oral Surgery and Implant Dentistry, Department of Oral and Maxillofacial Surgery, University Hospital of Geneva, 1205 Geneva, Switzerland; ^2^Department of Stomatology, Faculty of Dentistry, University of Seville, 41009 Seville, Spain; ^3^Department of Oral and Maxillofacial Surgery, Lausanne University Hospital (CHUV), 1011 Lausanne, Switzerland; ^4^Department of Surgical, Medical, Molecular and of Critical Area Pathologies, Complex Operative Unit of Stomatology and Oral Surgery, University-Hospital of Pisa, University of Pisa, 56126 Pisa, Italy

## Abstract

Tooth autotransplantation is a procedure which provides the extraction of an erupted or impacted tooth and its repositioning to another site in the oral cavity. This Case Report describes a successful case of two autotransplantations of open-apex mandibular third molars in place of the hopeless first mandibular molars with the use of L-PRF in a growing patient. A 15-year-old male patient was referred to the Dental Clinic for the extractions of the two hopeless mandibular first molars. Autotransplantation was considered the best treatment option for both sites 36 and 46, because the presence of two impacted mandibular third molars (38 and 48) with an incomplete root formation. Teeth 36 and 46 were extracted and replaced with teeth 38 and 48. The patient had an uneventful healing. At follow-up visits, the two autotransplanted teeth showed physiologic mobility, absence of inflammation and discomfort, and absence of infection; probing depth values were within normal range, and the vitality tests were positive. After 2 years, the teeth in position 36 and 46 showed absence of infection and mobility, and positive pulp vitality tests and the radiographic examinations exhibited closure of the root apices as well as absence of any periapical radiolucency or root resorption. Tooth autotransplantation is a good treatment option in case of tooth loss offering an alternative to traditional or implant-supported prosthesis especially for growing patients.

## 1. Introduction

Tooth autotransplantation is a procedure that involves extraction of an erupted or impacted tooth and its repositioning to another site within the same individual's oral cavity [1]. This treatment has shown several advantages and should always be taken in consideration together with other restorative treatment options such as removable or fixed restorative treatment, orthodontic treatment, and osseointegrated implants [2]. In contrast to a dental implant—which cannot be placed in those patients who have not completed the craniofacial skeletal growth—tooth transplantation is remarkably helpful in growing patients because it allows the tooth to maintain its function and proprioception and to follow the patient's facial growth pattern [3]. After a complete healing of the tooth transplantation, the preservation of its periodontal ligament allows orthodontic movement, if required. In addition, the pulp vitality of the transplanted tooth can be easily preserved in cases of open apex tooth; therefore, the donor tooth could undergo a complete root formation and growth [4]. In a long-term study, the authors revealed that the apical root foramen should be greater than 1 mm to have a predictable pulp revascularization [5]. On the contrary, teeth with closed apices had less frequent pulp healing and revascularization, thus, requiring endodontic treatment beforehand or at 2 weeks after tooth transplantation [6]. As in almost all surgical procedures, some complications have been reported in tooth transplantations, such as pulp necrosis, infections, root resorption, and ankylosis [7]. The scientific literature has reported various success and survival rates with a wide range of percentages that go from 60% to 95% [1, 3, 8]. Although at present no evidence-based parameters exist to evaluate the clinical success for autotransplantation, the following outcomes could be considered successful: complete healing for gingival and bone tissues, a restored tooth vitality, a tooth mobility within a physiological range, and an absence of root resorption. Recently, in a systematic review, some authors observed an average survival rate of 89.1% for transplanted teeth, showing that this procedure could be considered a reliable treatment option even when compared to single implants that had a survival rate of 96% [9].

Leukocyte-Platelet-Rich Fibrin (L-PRF) is an autologous blood-derived product containing a fibrin clot with serum and platelets, which enhances healing processes [10]. L-PRF is characterized by the absence of any additives in the blood sample and plays a role in improving wound healing processes by promoting cellular proliferation and angiogenesis [11]. Although many studies have focused on the use of L-PRF in oral surgical procedures such as impacted tooth extractions and bone grafting, there is still a lack of knowledge on its application in tooth autotransplantations.

This case report describes a successful case of autotransplantation of open-apex mandibular third molars in place of the first mandibular molars with the use of L-PRF and preservation of their vitality over 2 years of follow-up.

## 2. Case Report

**Figure 1:**
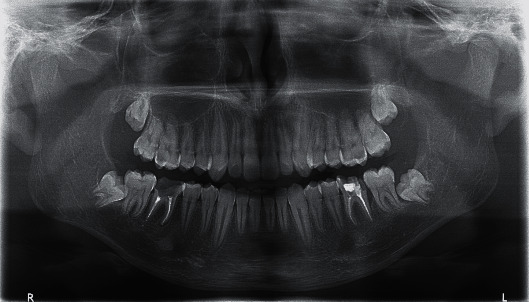
Initial panoramic radiograph.

A 15-year-old male patient in good general health was referred to the Dental Clinic at the University of Geneva (Switzerland) for the extractions of the two mandibular first molars (46 and 36) that were hopeless because of extensive caries. The two teeth had been endodontically treated, and both had shown perforation of the pulp chamber's floor due to decay, and, additionally, tooth 36 had shown a distal vertical bone defect. The patient's medical history was not-contributory, and an extensive clinical examination revealed poor oral hygiene and a few other carious lesions. The patient showed a full mouth plaque score > 30% and a full mouth bleeding score > 35%. To keep etiologic factors under control, the patient underwent scaling and received oral hygiene instructions. After evaluating multiple rehabilitative solutions, including orthodontic treatment and temporary partial removable prosthesis, autotransplantation was considered the best treatment option for both site 46 and 36. Teeth 38 and 48 were considered as donor teeth because they had open apices, not-completed root development and were impacted (Figure 1).

The patient's parents provided verbal and written informed consent after explanation and discussion of the treatment plan and before starting any procedures. The perioral skin and intraoral mucosa disinfection were performed with 0.1% chlorhexidine swab, and local anesthesia was administered using 4% articain solution for block and periapical anesthesia.

**Figure 2:**
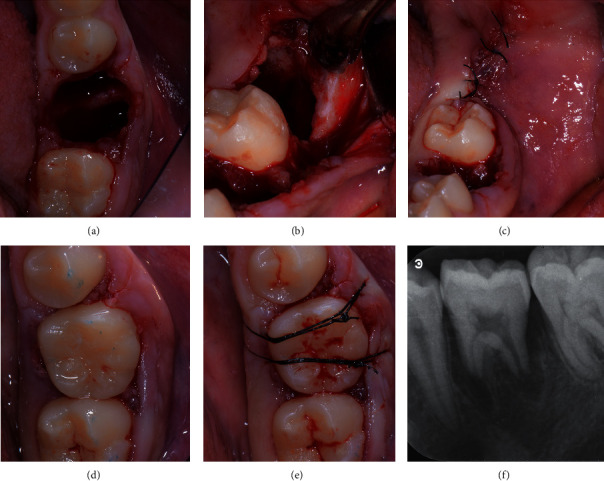
Tooth 36: (a) alveolar extraction socket; (b) alveolar extraction socket of left third molar; (c) sutures of third molar extraction site; (d) transplanted tooth in position 36; (e) cross-sutures for stabilization; (f) periapical radiograph immediately after transplantation.

**Figure 3:**
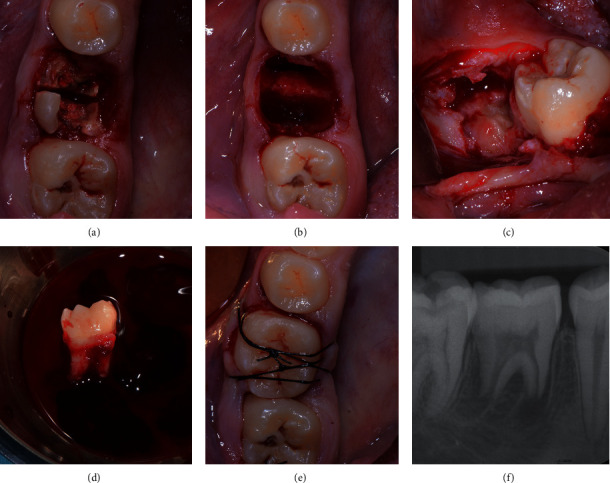
Tooth 46: (a) root's separation; (b) alveolar fresh extraction socket; (c) impacted right third molar; (d) third molar donor tooth extracted and stored in the L-PRF exudate; (e) transplanted tooth in position 46 and cross-sutures for stabilization; (f) periapical radiograph immediately after transplantation.

The first mandibular molars were extracted trying to reduce the trauma as much as possible. The roots were separated using high-speed fissure burs and were carefully luxated (Figures 2 and 3). The extraction sockets were thoroughly cleaned and irrigated with NaCl solution to remove all residual granulation tissues.

Platelet-Rich-Fibrin (L-PRF, IntraSpin®, Boca Raton, FL, USA) was prepared according to the manufacturer's guidelines. Four 9 ml tubes were filled with peripheral blood by venipuncture. The tubes were centrifuged at 2700 rpm using the IntraSpin® centrifuge for 12 minutes, the fibrin clots were transferred to the Xpression™ box (IntraSpin®, Boca Raton, FL, USA), and after 5 minutes, the L-PRF membranes were ready for use.

At the donor tooth sites, a full thickness envelope flap was raised, and bone removal was performed on the distal and buccal sites down to the cervical line of the tooth in order to completely expose the crown and make the tooth extraction as atraumatic as possible. The donor teeth were gently removed taking care to avoid any pressure and mechanical damage to the periodontal ligament. Once extracted, the donor teeth were stored in the L-PRF exudate, taken from the Xpression™ box, and the receiving sites were prepared by removing the interradicular bone. A minimal shaving of third molar crowns was necessary to adjust the mesiodistal width to the space available, and all efforts were taken to minimize tooth extraoral permanence to less than 7 minutes. The L-PRF membranes were placed in the receiving sites, and, subsequently, the mandibular third molars were placed in the first molar extraction sites. A good fit of the donor tooth into the receiving sites was achieved.

Sutures were placed across the occlusal surfaces to better stabilize the transplanted teeth and to reposition the flaps. The two transplanted teeth showed a good stability in their position, thus, not requiring any additional stabilization.

During the postoperative period, the patient was prescribed 2 grams of amoxicillin daily for 5 days, 1000 mg of paracetamol every 8 hours according to pain, and 0.12% chlorhexidine mouth-rinse for the following 4 weeks. The patient was also instructed to avoid brushing and interdental cleaning in the surgical areas for 4 weeks and to follow a soft diet for 2 weeks.

**Figure 4:**
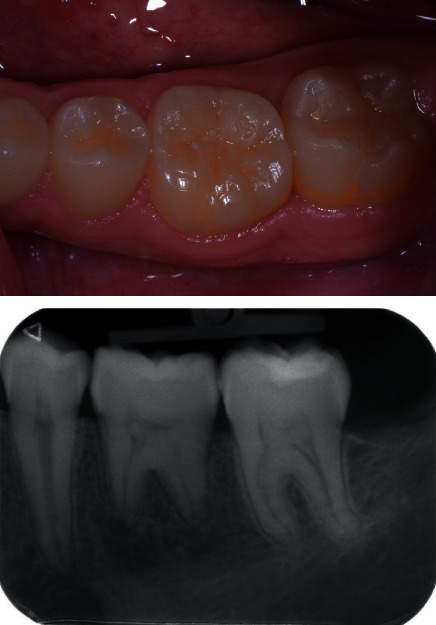
Tooth 36 at 1 year of follow-up.

**Figure 5:**
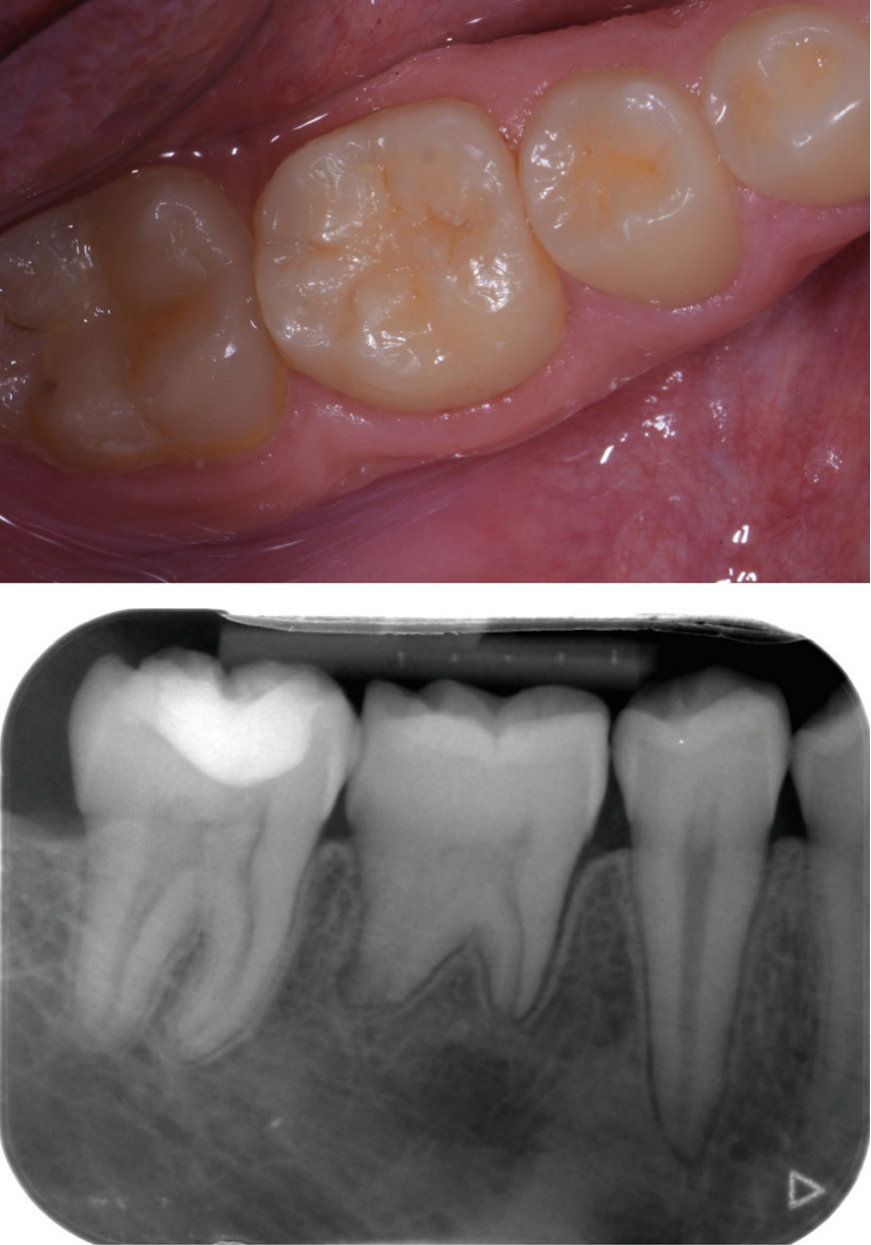
Tooth 46 at 1 year of follow-up.

**Figure 6:**
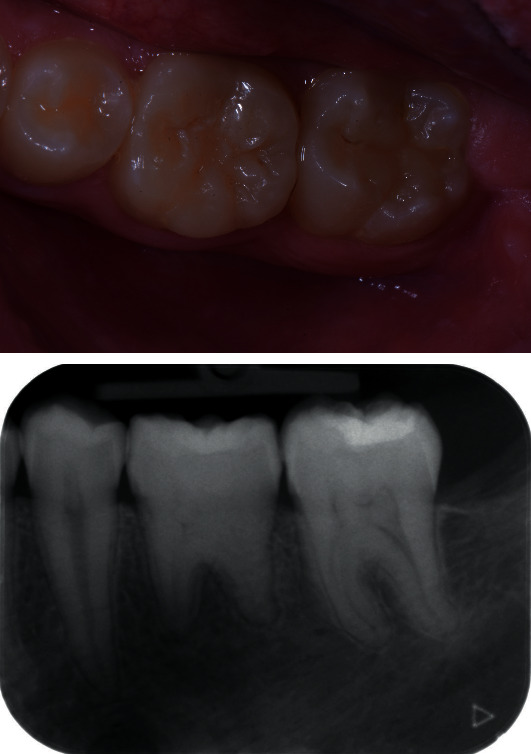
Tooth 36 at 2 years of follow-up.

**Figure 7:**
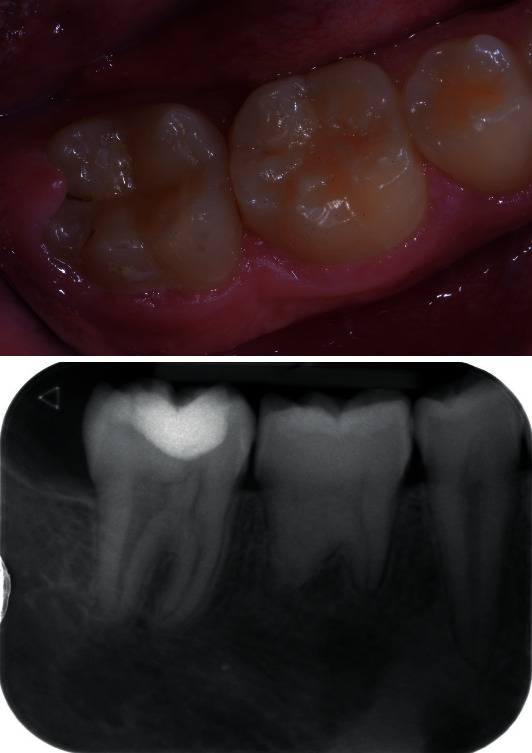
Tooth 46 at 2 years of follow-up.

**Figure 8:**
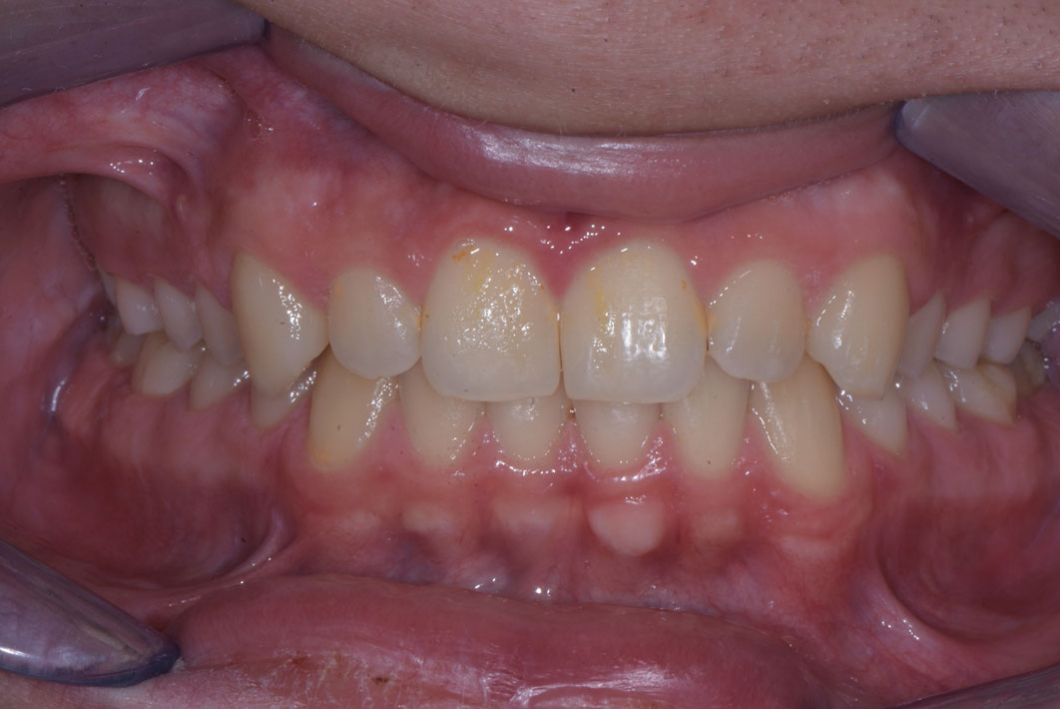
Clinical view at 2 years of follow-up.

Patient healing was uneventful, and the sutures were removed after 1 week. The decision to avoid endodontic treatment was based on the presence of open apices and undeveloped roots. A large consensus, based on reports and reviews, underlines that in presence of developing roots with open apices, the best practice is to allow for a natural pulp healing with a strict clinical monitoring. At 2-month follow-up, the clinical examination revealed that both autotransplanted teeth had physiologic mobility, absence of inflammation, and discomfort. At 6-month follow-up, the patient showed absence of inflammation or infection, probing depth values within normal range, and the vitality tests were positive for both autotransplanted teeth. After 1- (Figures 4 and 5) and 2-year (Figures 6–8) follow-up, the teeth in position 36 and 46 showed absence of infection and mobility, and positive pulp vitality tests and the radiographic examinations exhibited closure of the root apices and absence of any periapical radiolucency or root resorption. The patient reported satisfactory masticatory function and absence of pain, discomfort, or other adverse events.

## 3. Discussion

This report shows a successful case of two open apex third-molar autotransplantations that maintained their pulp vitality and function after 2 years of follow-up.

Tooth autotransplantation makes it possible to replace teeth with an unfavorable prognosis due to extensive carious lesions, tooth anomalies, and fractures, which can be caused by traumatic injuries. In the past years, some cases of oral piercing-associated tooth fractures were described, especially in young patients [12].

Dental implants are generally considered an ideal treatment option in case of missing teeth for patients with permanent dentition but a few clinical issues should be considered when this treatment is evaluated in young patients. First, dental implants are not free from biological and mechanical complications, and the longer the implants will be used, the higher the risk of complications could be. Second, the impact of facial growth and development on dental implants is well known as well as the risks of implant infraocclusion and displacement especially for implants in the esthetic area. Therefore, the autotransplantation should be considered a viable option because it can be performed in young growing patients, when implants are fully contraindicated, as it allows the maintenance of periodontal ligaments, and, consequently, the transplanted tooth can follow the facial growth pattern and can be moved orthodontically. An important consideration is the cost-effectiveness of autotransplantation: the treatment cost for an autotransplantation is definitely lower than for an implant rehabilitation. This is fully in agreement with a retrospective study where the authors have reported that the choice for the autotransplantation approach was based on the economic cost evaluation in 35% of patients [13].

Some recent reviews and meta-analyses have found that the overall survival and success rates for the autotrasplantation approach were high and ranged between 75% and 95%. Although the percentages of success are very high, a careful analysis of the scientific data could reveal a remarkable variety of the success/survival rates. Indeed, the discordance in the clinical outcomes presumably indicates a wide variety between the different scientific reports. Despite the amount of studies present in the scientific literature, the quality of evidence is still low because of methodological limitations and technical discrepancies among the different studies. Moreover, it has been clearly shown that multirooted teeth like molars have a significantly less favorable prognosis than single rooted teeth [14].

Several criteria have been observed as key factors for obtaining a successful autotrasplantation. The recipient sites have to be free from infections and need to have enough bone to allow an adequate stabilization for the transplanted tooth. In terms of donor sites, the teeth with uncomplete root development are the ideal candidates for transplantation because they have potential for root formation and maintained pulp vitality. Some other prognostic factors that could impact the success rates of autotransplantation are the atraumatic tooth extraction, the limitations of injuries of the root and of the periodontal ligament, minimal handling of the root, and reduced extraoral storage time. All the above reported factors are associated with minimizing the risk for periodontal ligament damage, which would predispose to the most frequent complications reported with autotransplants such as internal/external root resorption and ankylosis.

L-PRF has been claimed to promote wound healing processes and angiogenesis [10]. In the present case, the use of L-PRF at donor sites may have improved the natural process of revascularization of the transplanted teeth. Moreover, the storage of the extracted teeth in the L-PRF exudate may have contributed to the maintenance of viability of the cells of both pulp and periodontal ligament, improving the clinical outcomes.

However, the overall cumulative occurrence of complications appears to be extremely nonconsistent among different studies; ankylosis, for instance, was reported to be 40% in some studies [14] and 0% in some others [13]. Recently, a study showed that a radiographic-guided approach with the use of a replica would significantly improve the predictability of the treatment when compared to a conventional approach [15]. The hypothesis was that the use of a surgical tooth-replica to shape the receiving site could significantly decrease the risk of damaging the periodontal ligament of donor tooth and, therefore, could increase the success/survival rates of autotransplanted teeth.

The autotransplantation is a good treatment option in case of tooth loss especially for young and/or growing patients, in which dental implants are contraindicated. Moreover, the use of L-PRF in our clinical case seems to have played a beneficial role in the surgical procedure outcomes.

The absence of a good level of scientific evidence should encourage randomized controlled studies to better evaluate the usefulness of this procedure.
